# Text-based phenotypic profiles incorporating biochemical phenotypes of inborn errors of metabolism improve phenomics-based diagnosis

**DOI:** 10.1007/s10545-017-0125-4

**Published:** 2018-01-16

**Authors:** Jessica J. Y. Lee, Michael M. Gottlieb, Jake Lever, Steven J. M. Jones, Nenad Blau, Clara D. M. van Karnebeek, Wyeth W. Wasserman

**Affiliations:** 1 Centre for Molecular Medicine and Therapeutics, BC Children’s Hospital Research Institute, University of British Columbia, Room 3109, 950 West 28th Avenue, Vancouver, BC V5Z 4H4 Canada; 2 Canada’s Michael Smith Genome Sciences Centre, BC Cancer Agency, Vancouver, BC Canada; 3 Department of Medical Genetics, University of British Columbia, Vancouver, BC Canada; 4 Dietmar-Hopp Metabolic Center, Department of General Pediatrics, University Hospital, Heidelberg, Germany; 5 Department of Pediatrics, University of British Columbia, Vancouver, BC Canada; 6 Departments of Pediatrics and Clinical Genetics, Emma Children’s Hospital, Academic Medical Centre, Amsterdam, The Netherlands

**Keywords:** Biochemical phenotypes, Metabolic phenotypes, Clinical informatics, Text-based phenomics, Data mining, Inborn errors of metabolism

## Abstract

Phenomics is the comprehensive study of phenotypes at every level of biology: from metabolites to organisms. With high throughput technologies increasing the scope of biological discoveries, the field of phenomics has been developing rapid and precise methods to collect, catalog, and analyze phenotypes. Such methods have allowed phenotypic data to be widely used in medical applications, from assisting clinical diagnoses to prioritizing genomic diagnoses. To channel the benefits of phenomics into the field of inborn errors of metabolism (IEM), we have recently launched IEMbase, an expert-curated knowledgebase of IEM and their disease-characterizing phenotypes. While our efforts with IEMbase have realized benefits, taking full advantage of phenomics requires a comprehensive curation of IEM phenotypes in core phenomics projects, which is dependent upon contributions from the IEM clinical and research community. Here, we assess the inclusion of IEM biochemical phenotypes in a core phenomics project, the Human Phenotype Ontology. We then demonstrate the utility of biochemical phenotypes using a text-based phenomics method to predict gene-disease relationships, showing that the prediction of IEM genes is significantly better using biochemical rather than clinical profiles. The findings herein provide a motivating goal for the IEM community to expand the computationally accessible descriptions of biochemical phenotypes associated with IEM in phenomics resources.

**Electronic supplementary material:**

The online version of this article (10.1007/s10545-017-0125-4) contains supplementary material, which is available to authorized users.

## Introduction

Patient phenotyping marks the beginning of the fundamental process of clinical genetics: uncovering the genetic etiology of the disease. The rate of genetic discovery has been accelerated by the adoption of genome-wide sequencing, and continues to generate an explosive amount of compiled phenotypic and genetic information (Chong et al 2015; Amberger et al 2011). Such abundance is motivating increasingly sophisticated efforts to (i) define a new phenotype and (ii) distinguish a novel phenotype from an existing one (Biesecker 2004; Amberger et al 2011). Therefore, both the scientific and clinical communities have focused on the acquisition of precise and comprehensive phenotypic data, or “phenomics” (Brunner and van Driel 2004; Houle et al 2010; Hennekam and Biesecker 2012; Robinson 2012; Deans et al 2015).

Scientifically, the word “phenome” refers to the entirety of observable traits from all levels of the biological hierarchy: from metabolites to organisms (Houle et al 2010). Clinically, the word refers to a collection of morphological, physiological, and behavioral characteristics observed in a patient (Robinson 2012). In either context, the field has seen numerous developments of large-scale projects (Houle et al 2010; Amberger et al 2015; Mungall et al 2017; Blake et al 2017). A successful example of such is the widely used Human Phenotype Ontology (HPO), which provides a standardized vocabulary of abnormal phenotypes observed in human diseases (Köhler et al 2017). HPO illustrates the value and motivation behind phenomics: (i) it enables accurate and consistent description of phenotypes, and (ii) it enables computational assessment of similarity between phenotypes (Köhler et al 2017). Based on the two attributes, HPO has become a foundation for computational methods that collect (Girdea et al 2013), catalog (Mungall et al 2017), share (Gottlieb et al 2015; Philippakis et al 2015), and analyze (Köhler et al 2009) phenotypic data. Furthermore, it has been demonstrated that precise, comprehensive profiling and analysis of phenotypes using HPO can augment clinical exome/genome sequencing data interpretation (Bone et al 2016; Sifrim et al 2013; Smedley and Robinson 2015).

However, phenomics has not yet been fully exploited in some domains of rare genetic diseases (Boycott et al 2017; Köhler et al 2017). Inborn errors of metabolism (IEM) exemplify one such domain (Köhler et al 2017). Caused by genetic defects in metabolism, IEM represent the largest group of monogenetic defects that are amenable to targeted treatments (Tarailo-Graovac et al 2016). They present distinct biochemical phenotypes and a heterogeneous array of clinical symptoms (Burton 1998). This characteristic has motivated the IEM clinical and research community to document both clinical and biochemical aspects of IEM (Lee et al 2017). Meanwhile, recent developments in phenomics have focused primarily on clinical aspects (Köhler et al 2017), resulting in an underrepresentation of biochemical phenotypes that may have slowed the uptake of phenomics by the IEM community. Moreover, deep phenotyping has become increasingly important for IEM as genome-wide sequencing identifies a growing number of cases with two distinct genetic diseases that present blended phenotypes (Tarailo-Graovac et al 2016). To address this gap, we created IEMbase, an expert-curated knowledgebase of IEM and their phenotypes (Lee et al 2017). However, our efforts only partially fill the gap, and the need for concurrent curation of IEM phenotypes in core phenomics projects remains.

Thus, we assessed the curation status of IEM phenotypes in HPO in comparison with IEMbase. We then extracted disease-characterizing phenotypic data from IEMbase and demonstrated their utility in diagnostic applications of phenomics using a text-based method that prioritizes compatible genetic diagnoses. We hope the findings presented herein catalyze community-wide participation to accelerate the cataloging of IEM phenotypes in IEMbase and HPO.

## Methods

The methods presented herein require a distinction between biochemical and clinical phenotypes of IEM. We define biochemical phenotypes as biochemical abnormalities that are observable by laboratory investigations. We define clinical phenotypes as morphological, (patho-)physiological, developmental, and behavioral abnormalities observable by clinical examinations.

## Assessment of biochemical phenotype curation in HPO and IEMbase

We previously compiled the clinical aspect of IEM and explored their representation within HPO (Lee et al 2017). Therefore, only the biochemical aspect of IEM was the focus of this effort. In the aforementioned study, we were not able to map biochemical phenotypes in IEMbase to HPO due to the stringent criteria requiring exact character-by-character matches. Based on this knowledge, the comparison presented herein used relaxed criteria.

**Fig. 1:**
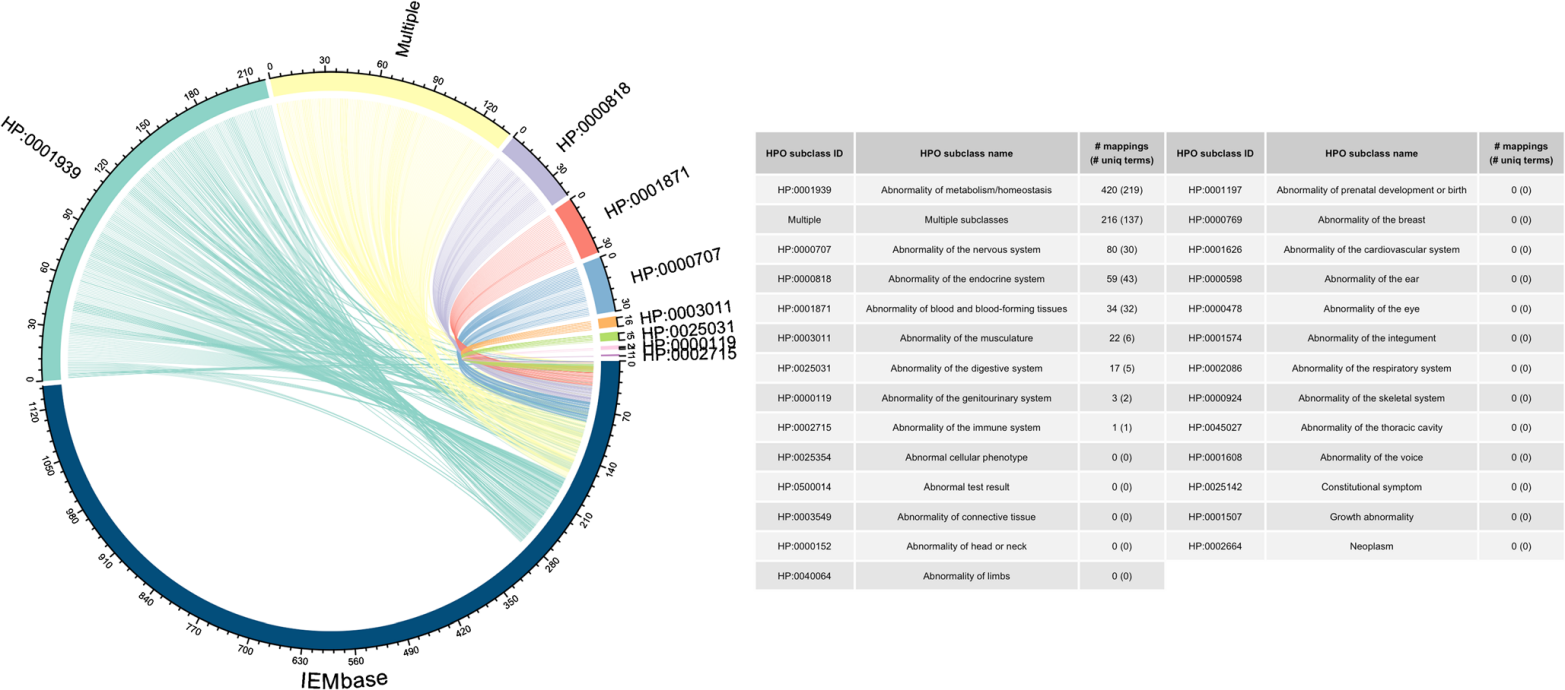
An overview of HPO to IEMbase mapping. 287 biochemical phenotypes in IEMbase had 852 associations with 475 unique HPO phenotypes. The figure illustrates such mappings with respect to 26 subclasses of the HPO class “phenotypic abnormality (HP:0000118)”. “Multiple subclasses” refer to HPO phenotypes that belong to multiple subclasses, consisting of: abnormality of metabolism/homeostasis (HP:0001939), abnormality of the genitourinary system (HP:0000119), abnormality of the endocrine system (HP:0000818), abnormality of the nervous system (HP:0000707), abnormality of blood and blood-forming tissues (HP:0001871), abnormality of the immune system (HP:0002715), and abnormality of the digestive system (HP:0025031)

For this assessment, a complete list of phenotypes in HPO was downloaded from the HPO website (http://human-phenotype-ontology.github.io) in OBO format (version: 2017–06-30 release). Using the ontologyIndex R package (Greene et al 2017) (R version 3.4.0), the OBO file was parsed, and all phenotypes and their synonyms pertaining to “phenotypic abnormality (HP:0000118)” were extracted (*n* = 37,732). In parallel, a complete list of phenotypes in IEMbase was downloaded from the IEMbase server (version: 1.1.0) in CSV format. The downloaded list contained 1151 biochemical phenotypes and 1231 clinical phenotypes. Only the biochemical phenotypes were extracted for the assessment. Before comparing the two, differences in alphabetic case, singular/plural variants, punctuation, stop words, and word order were removed using the Norm program in the SPECIALIST Lexical Tools (Browne et al 2003). The HPO phenotypes were then compared against the IEMbase phenotypes using a custom script written in Ruby programming language. A match was declared only if the name of a HPO phenotype had an exact match or it completely contained the name of an IEMbase phenotype. As an example of the latter, the HPO phenotype “elevated urinary homovanillic acid (HP:0011977)” was considered a match for the IEMbase phenotype “homovanillic acid” since the HPO phenotype contained both the word “homovanillic” and the word “acid”. After the computational comparison, the phenotype matches were reviewed manually. The mappings were then grouped by their membership in the 26 subclasses of the HPO class “phenotypic abnormality (HP:0000118)”. A detailed list of the 26 subclasses is provided in Fig. 1. Finally, the grouped mappings were visualized in a Circos plot using the circlize R package (Gu et al 2014).

## Text-based phenotype analysis for prioritization of causal genes

**Fig. 2:**
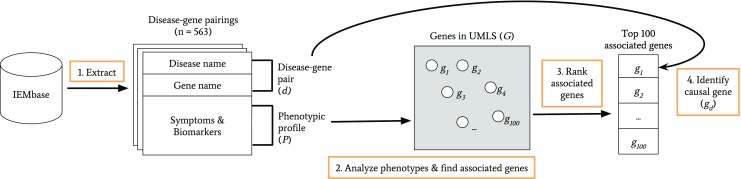
An illustration of the text-based phenotype analysis procedure. Numbered boxes (in orange) represent the main steps of the text-based phenotype analysis. First, 563 disease-gene pairings were extracted from IEMbase (v. 1.1.0). Each pair contained the disorder name and gene name, and the pair was coupled to a phenotypic profile (i.e., disease symptoms and biomarkers). Second, using the phenotypic profile *P*, associated genes were identified using a text-analysis tool by Lever et al. The association strength between *P* and *g* was defined as the ratio of the number of sentences in the PubMed literature where *P* and *g* appeared together over the total number of sentences where *P* and *g* appeared individually. Third, the identified genes were ranked by the strength of their association with *P* before a list of top 100 associated genes was determined. Finally, the causal gene *g*_*d*_ was identified based on the disease-gene pair connected to *P*. The rank of *g*_*d*_ was recorded

Figure 2 illustrates the analysis procedure. Five hundred sixty-three disease-gene pairings (or “pairs”) and their phenotypic descriptions (or “profiles”) were downloaded from the IEMbase server (version: 1.1.0). An example disease-gene pair and its phenotypic profile are provided in Table 1. In order to apply the text-based phenotype analysis described in the next paragraph, the phenotypes in each profile were equated to the corresponding terms in the Unified Medical Language System (UMLS) (https://www.nlm.nih.gov/research/umls) using the UMLS REST API (https://documentation.uts.nlm.nih.gov/rest/home.html). For clarity, the mapping between IEMbase and HPO from the earlier section does not relate to the mapping exercise described herein.

**Table 1:** An example disease-gene pair and its phenotypic profile extracted from IEMbase

Disease name	Dopamine beta-hydroxylase deficiency
Associated gene	*DBH*
Phenotypes*	Exercise intolerance
	Hypoglycemia
	Hypotension, orthostatic
	Dopamine (plasma)
	Epinephrine (plasma)
	Homovanillic acid, HVA (cerebrospinal fluid)
	Vanillinmandelic acid, VMA (urine)

*Only select phenotypes are listed for brevity

Each phenotypic profile was analyzed using a text-based method that was originally developed for variant prioritization in clinical exome interpretation (Gottlieb 2017). Briefly, the method accepts a set of phenotype terms and returns a ranked list of genes. The ranking was calculated based on information reported by a text analysis system (Lever et al 2017). For our analysis, the procedure was performed as follows. A disease-gene pair *d* was selected from the set of all IEMbase disease-gene pairs *D* = {*d*_1_, *d*_2_, …, *d*_*n*_}. Within IEMbase *d* was coupled to a phenotypic profile *P*, which contained a set of phenotypes {*p*_1_, *p*_2_, …, *p*_*r*_} as illustrated in Table 1. The method then predicted associated genes for *P* from the genome *G* = {*g*_1_, *g*_2_, …, *g*_*m*_} which was defined as all genes pertaining to the UMLS semantic type “gene or genome (T028)”. For each *g* ∈ *G*, the strength of its association with *P* (denoted by *s*_*g*, *P*_) was determined as a sum of individual association scores between *g* and *p*_*i*_. The individual association score was calculated as the ratio of the number of sentences where *g* and *p*_*i*_ appeared together over the total number of sentences where *g* and *p*_*i*_ appeared individually (where these values were obtained from the text analysis tool (Lever et al 2017)). Each gene *g* was ranked according to *s*_*g*, *P*_ and the top 100 phenotype *P*-associated genes were retained before the method continued on to the next disease *d* ∈ *D*.

For each *d*, the top 100 associated gene predictions were obtained using the method outlined above, and the rank of *d*’s causal gene *g*_*d*_ in the top 100 predictions was determined. To assess the performance of the text-based method, the ranking of all causal genes ${G}_d=\left\{{g}_{d_1},{g}_{d_2},\dots, {g}_{d_n}\right\}$ was compared against the baseline ranking of *G*_*d*_. The baseline ranking was defined as the median ranking of each *g*_*d*_ ∈ *G*_*d*_, which was determined by taking the median of *g*_*d*_’s ranks in the predictions for *d* ∈ *D* that *g*_*d*_ did not have a causal relationship with.

Furthermore, the effect of the number of phenotypes specified for each *d* ∈ *D* on its causal gene prediction was evaluated by testing their correlation. This test was restricted to only *d* ∈ *D* whose causal gene *g*_*d*_ was ranked within the top 100 predictions.

In addition, we assessed the impact of biochemical phenotypes for the disease gene prediction compared with clinical phenotypes. For this, the set of phenotypes *P* for each *d* ∈ *D* was divided into biochemical and clinical subsets, and each subset was then analyzed using the aforementioned text-based method to predict the top 100 associated genes. Finally, a comparison was made between the ranks of causal genes determined using biochemical phenotypes and the ranks determined using clinical phenotypes.

## Results

### Comparison of curated biochemical phenotypes between HPO and IEMbase

The curated IEMbase (v. 1.1.0) provides a total of 1151 biochemical phenotypes, of which only 287 could be mapped onto HPO. These 287 IEMbase biochemical phenotypes had 852 associations with 475 unique HPO phenotypes, indicating a one-to-many relationship between IEMbase and HPO. Figure 1 provides a visual overview of these mappings, which highlights the IEMbase biochemical phenotypes that map most commonly onto the HPO metabolism category (HP:0001939) (420 mappings to 219 unique phenotypes). A survey of 864 unmapped IEMbase biochemical phenotypes revealed that the majority were complex names, such as “7-alpha-hydroxy-3-oxo-cholenoic acids”. These unmapped phenotypes will be submitted to HPO for consideration for future inclusion.

### Evaluation of phenotype-associated gene predictions by text-based phenotype analysis

Using all phenotypes (biochemical and clinical), the text-based phenotype analysis prioritized correct genetic diagnoses for 120 out of 563 disease-gene pairs within the top ten predictions and 173 out of 563 disease-gene pairs within the top 20 predictions (Table 2). This performance was statistically assessed by comparing the causal gene ranking against the baseline ranking using the McNemar’s test (mcnemar.exact implemented by exact2x2 R package; Fay 2010) with the Bonferroni correction. A dichotomous trait for the McNemar’s test was defined as (1) disease-gene pairs whose causal genes ranked within the top N predictions or (2) disease-gene pairs whose causal genes did not rank within the top N predictions where *N* = 1, 5, 10, 20, 100. This assessment confirmed that the method placed causal genes within the top N predictions significantly more often than the baseline (Table 2). However, the method’s performance appeared to be limited as diagnoses for 255 disease-gene pairs were not found within the top 100 predictions (Table 2). This may be due to the inconsistent depth of literature on genes limiting the performance of the recommendation system as well as the lack of semantic representation in sentence-level co-occurrence. As an example of the latter, if a sentence in a publication described that “mutations in the gene *PAH* cause elevated blood phenylalanine”, then the phenotype-gene association was established based only on the co-occurrence of the words “*PAH*” and “phenylalanine” and not based on the fact that “phenylalanine” was “elevated” due to a defect in “*PAH*”.

**Table 2:** A summary of text-based phenotype analysis performance

N	Top 1	Top 5	Top 10	Top 20	Top 100
Number of disease-gene pairs ranked within top N predictions (% success at N)^a^	31 (5.5)	90 (16.0)	120 (21.3)	173 (30.7)	308 (54.7)
McNemar’s test at N (causal vs baseline) ^b^	*p* < 0.001	p < 0.001	p < 0.001	*p* < 0.001	p < 0.001

^a^% Success at N refers to the proportion of IEMbase disease-gene pairs whose causal genes ranked within the top N predictions

^b^McNemar’s test at N refers to paired comparison between the causal ranking and the baseline ranking with a dichotomous trait defined as (1) disease-gene pairs whose causal genes ranked within the top N predictions or (2) disease-gene pairs whose causal genes did not rank within the top N predictions where N = 1, 5, 10, 20, 100. Reported *p*-value was adjusted using the Bonferroni correction

Meanwhile, there was no significant effect on the causal gene predictions made by the number of phenotypes specified for the disease-gene pairs (*p* = 0.15; cor. test on Spearman’s correlation in R; Fig. S1 in Supplemental material).

**Fig. 3:**
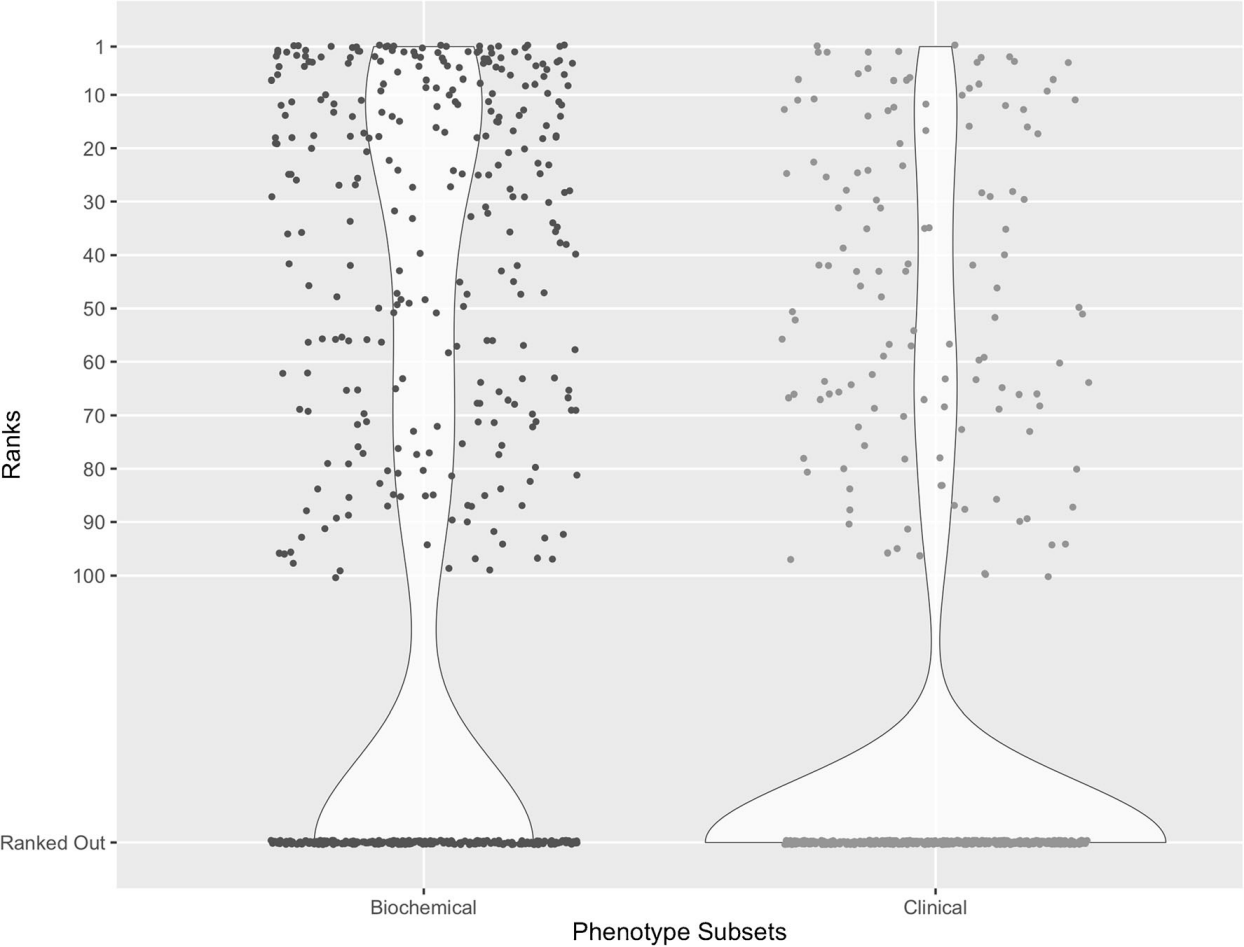
Distribution of ranks using only biochemical phenotypes vs using only clinical phenotypes. The x-axis represents the subset of phenotypes (biochemical-only and clinical-only). The y-axis represents the ranks of causal genes in the top N predictions. The distribution of ranks is shown in a violin plot (hour-glass figure). A scatter plot version of the same distribution (dot) is overlaid on top of the violin plot to show the position of each data point in the distribution. The text-based method predicted significantly more causal genes within the top N predictions (*N* = 1, 5, 10, 20, 100) using biochemical phenotypes than clinical phenotypes (Table 3; McNemar’s test with Bonferroni correction)

In the evaluation of the impact on gene predictions by biochemical phenotypes versus clinical phenotypes, significantly more causal genes were predicted within the top N predictions (N = 1, 5, 10, 20, 100) using biochemical phenotypes than clinical phenotypes (Table 3; McNemar’s test with Bonferroni correction). This result may suggest that the association between biochemical phenotypes and IEM genes are likely more represented in the current literature than clinical phenotypes and IEM genes. Figure 3 illustrates the difference in gene prediction performance between the two subsets of phenotypes.

**Table 3:** An overview of impact on gene predictions by biochemical phenotypes vs clinical phenotypes

N	Top 1	Top 5	Top 10	Top 20	Top 100
Number of disease-gene pairs ranked within top N predictions based on biochemical phenotypes (% success at N)^a^	19 (3.4)	67 (11.9)	88 (15.6)	132 (23.4)	292 (51.9)
Number of disease-gene pairs ranked within top N predictions based on clinical phenotypes (% success at N)^a^	2 (0.4)	12 (2.1)	22 (3.9)	37 (6.6)	132 (23.4)
McNemar’s test at N (biochemical vs clinical) ^b^	*p* = 0.0011	p < 0.001	p < 0.001	p < 0.001	p < 0.001

*Success at N refers to the proportion of IEMbase disease-gene pairs whose causal gene ranked within the top N predictions

^b^McNemar’s test at N refers to paired comparison between the biochemical ranking and the clinical ranking with a dichotomous trait defined as (1) genes ranked within the top N predictions or (2) genes not ranked within the top N predictions where N = 1, 5, 10, 20, 100. Reported p-value was adjusted using the Bonferroni correction

## Discussion

In this report, we explored and extended the utility of curated disease annotations for IEM for the emerging age of phenomics analysis. We assessed the overlap between biochemical phenotypes compiled by curators of IEMbase and all phenotypes within the HPO, noting limited coverage. We demonstrated that the use of biochemical phenotypes can significantly improve the prediction of gene-disease relationships for IEM, compared to clinical phenotypes, using text-based phenotype analysis.

The comparison of curated biochemical phenotypes between IEMbase and HPO revealed that only 25% of the biochemical phenotypes in IEMbase could be mapped to HPO. Incomplete mapping could arise for a number of reasons. For instance, (1) a HPO phenotype may not share the exact wording of the synonymous IEMbase phenotypes or (2) a more general HPO phenotype may refer to one or more specific IEMbase phenotypes. This suggests that future curation could significantly improve phenotype mapping, and contributions from the IEM clinical and research community would prove instrumental to increasing the utility of available phenotypic data. In addition, a collaboration between IEMbase and HPO to include missing terms can contribute to improved coverage of biochemical phenotypes in HPO.

The text-based phenotype analysis using all (biochemical and clinical) phenotypes revealed that genetic diagnoses for 31% of input disease-gene pairs could be successfully prioritized within the top 20 predictions. This number is too low for immediate diagnostic utility. However, mapping patient phenotypes to candidate genes would normally consider a richer set of information than just phenotypic descriptions. For example, in clinical exome/genome sequencing a comprehensive patient profile is constructed based on both clinical and laboratory investigations before prioritizing and interpreting a small set of genes containing genetic alterations (Tarailo-Graovac et al 2016; Bone et al 2016; Smedley and Robinson 2015). Therefore, the diagnostic utility of phenotypic data lies in its synergy with different investigative tools rather than its lone capacity to assist diagnoses.

The evaluation of text-based disease gene predictions showed better performance when incorporating biochemical phenotypes compared to clinical phenotypes. This difference could be explained by the non-specific and heterogeneous nature of clinical phenotypes of IEM (Leonard and Morris 2006). Such limitations have been recognized by the IEM community and have motivated the extensive use of biochemical tests in diagnoses (Tebani et al 2016). Given the IEM community’s emphasis on biochemical phenotypes, finding ways to accelerate the compilation of such annotations in IEMbase and to extend the inclusion of biochemical phenotypes in HPO are important in the near term to fully benefit from emerging advances in phenomics. An expanded curation of phenotypes in HPO can improve recognition of heterogeneous disease presentations and overlapping phenotypes in text-based phenotype analyses, as the performance of such methods are limited by the availability of curated disease annotations. In the future, as HPO expands, curation efforts can provide greater granularity of biochemical phenotypes by incorporating either continuous measurements or levels relative to clinical decision criteria.

For readers who would like to contribute to data curation, IEMbase accepts submissions of new or expanded IEM phenotypes, as well as edit requests to currently curated information, via the project website (http://iembase.org/app). HPO accepts new term submissions via an issue tracker available on Github (https://github.com/obophenotype/human-phenotype-ontology/issues). To submit a term to HPO, please consult the submission guideline (https://github.com/obophenotype/human-phenotype-ontology/wiki/How-to-make-a-good-term-request) and create an issue using the “New issue” button on the issue tracker page.

In summary, there is synergistic utility in phenotypic data of IEM and phenomics methods that could be harnessed by a multitude of diagnostic methods. With the imminent shift toward a holistic clinical investigation using multi-omics technologies (such as metabolomics, lipidomics, and glycomics), we believe that a comprehensive knowledgebase of phenotypes will serve as the basis upon which different layers of data are integrated. Before realizing such a role, however, the knowledgebase must ensure complete incorporation of HPO into its structure in order to accommodate the complexity of the upcoming big phenotypic data. As such, community-wide efforts for curation of biochemical phenotype data should be recognized as a critical step toward precision medicine.

## Electronic supplementary material

ESM 1 (DOCX 255 kb)
