# Supplementary material for: Text-based phenotypic profiles incorporating biochemical phenotypes of inborn errors of metabolism improve phenomics-based diagnosis
Source: J Inherit Metab Dis. 2018 Jan 16;41(3):555–62. doi: 10.1007/s10545-017-0125-4 (PMC5959948; doi:10.1007/s10545-017-0125-4)
Supplement: Supplementary file 1 — (DOCX 255 kb) [file 10545_2017_125_MOESM1_ESM.docx]

**Supplementary Material**

**Figure S1. Correlation between the number of phenotypes and the rank of top 100 predicted genes (p=0.15; cor.test in R on Spearman’s correlation).**

**
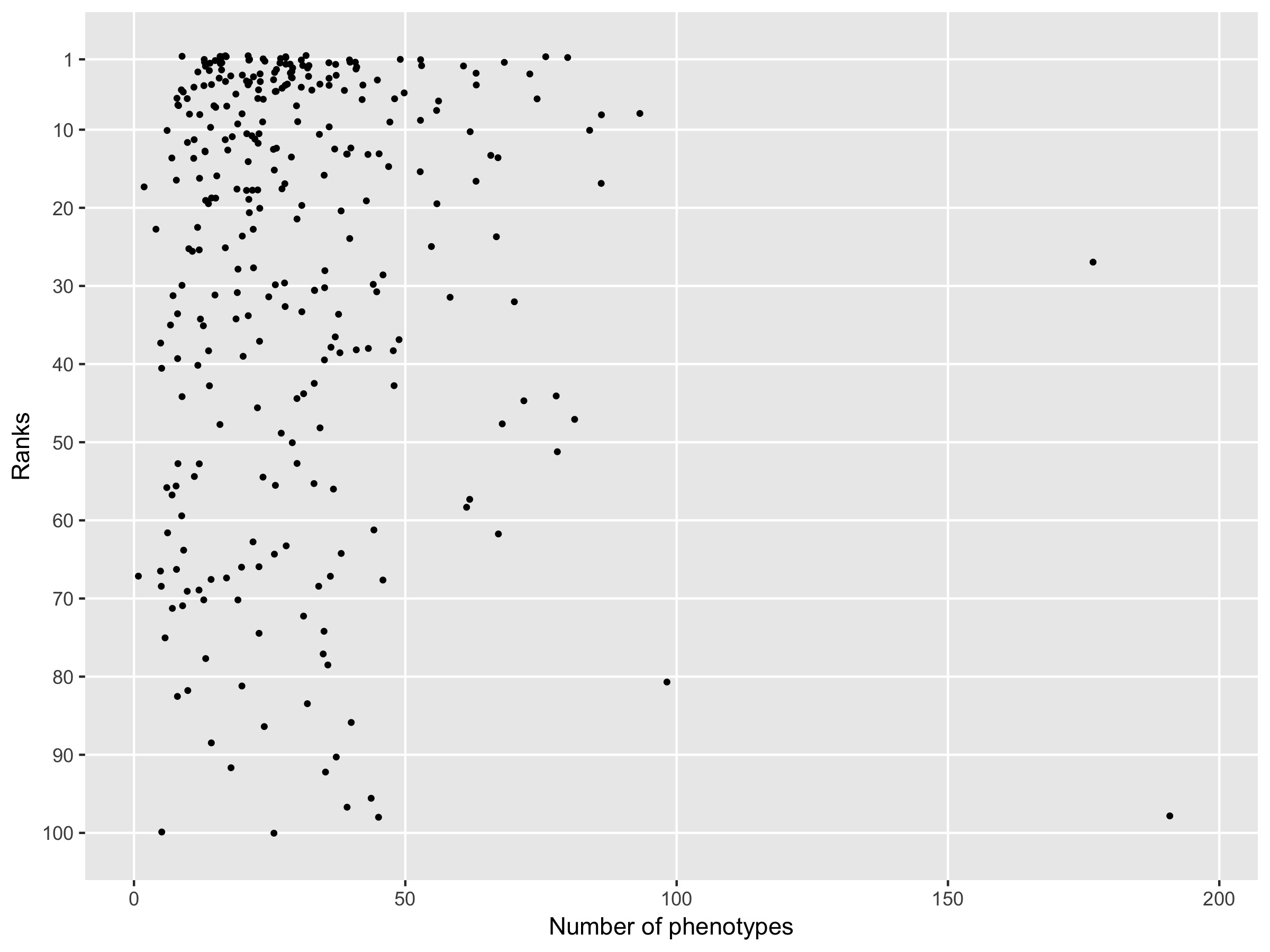
**

No significant correlation was found between the number of phenotypes and the rank of top 100 predicted genes. The correlation analysis was performed to test whether the gene predictions were associated with the number of phenotypes.
